# Dynamic changes of IgM and IgG antibodies in asymptomatic patients as an effective way to detect SARS‐CoV‐2 infection

**DOI:** 10.1002/jcla.24080

**Published:** 2021-12-16

**Authors:** Ping Li, Ge Shen, Zhenhua Zhu, Shengjie Shi, Yan Hu, Ziyan Zeng, Hui Zhou, Qiong Li, Pan Zhu, Gang Yang, Zugui Liu, Huiyuan Fu, Junyu Hu, Ying He, Qingting Yang, Miao Dai, Dan Zhou, Qingqing Lu, Xiaobing Xie

**Affiliations:** ^1^ Medical Laboratory Center First Hospital of Hunan University of Chinese Medicine Changsha China; ^2^ Loudi Center for Diseases Control and Prevention Loudi China; ^3^ Department of Otorhinolaryngology First Hospital of Hunan University of Chinese Medicine Changsha China

**Keywords:** asymptomatic, COVID‐19, IgG, IgM, SARS‐CoV‐2

## Abstract

**Background:**

COVID‐19 has become a global pandemic, and close contacts and asymptomatic patients are worthy of attention.

**Methods:**

A total of 1844 people in close contacts with 76 COVID‐19 patients were investigated, and nasopharyngeal swabs and venous blood were collected for centralized medical quarantine observation. Real‐time fluorescence was used to detect SARS‐CoV‐2 nucleic acid in nasopharyngeal swabs of all close contacts, and the colloidal gold method was used to detect serum‐specific antibodies. Levels of IgM‐ and IgG‐specific antibodies were detected quantitatively through chemiluminescence from the first nucleic acid turned negative date (0 week) and on weekly intervals of ≤1 week, 1–2 weeks, 2–3 weeks, 3–4 weeks, 4–5 weeks, 5–6 weeks, and 6–7 weeks.

**Results:**

The total positive rate of the colloidal gold method (88.5%, 23/26) was significantly higher (χ^2^ = 59.182, *p* < 0.001) than that of the healthy control group (2.0%, 1/50). There was significant difference in IgG concentration at different time points (0–7 weeks) after negative nucleic acid conversion (χ^2^ = 14.034, *p* = 0.029). Serum IgG levels were significantly higher at weekly time points of 4–5 weeks (Z = −2.399, *p* = 0.016), 5–6 weeks (Z = −2.049, *p* = 0.040), and 6–7 weeks (Z = −2.197, *p* = 0.028) compared with 1–2 weeks after negative nucleic acid conversion. However, there was no significant difference (χ^2^ = 4.936, *p* = 0.552) in IgM concentration between time points tested (0–7 weeks) after negative nucleic acid conversion. The positive rates of IgM and IgG in asymptomatic patients (χ^2^ = 84.660, *p* < 0.001) were significantly higher than those in the healthy control group (χ^2^ = 9.201, *p* = 0.002) within 7 weeks of negative nucleic acid conversion.

**Conclusions:**

The IgG concentration in asymptomatic cases remained at a high level after nucleic acid turned negative. Nucleic acid detection combined with IgM and IgG antibody detection is an effective way to screen asymptomatic infections.

## Graphical abstract

COVID‐19 has become a global epidemic, close contacts and asymptomatic patients are worthy of attention. A total of 1844 people in close contact with 76 COVID‐19 patients were investigated, and nasopharyngeal swabs and venous blood were collected for centralized medical quarantine observation. The IgG concentration in asymptomatic cases remained at a high level after nucleic acid turned negative. Nucleic acid combined with IgM and IgG antibody detection is a more effective way to screen asymptomatic infections.

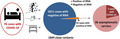

## 1 INTRODUCTION

In December 2019, an unexplained viral pneumonia was first reported in Wuhan, Hubei Province, China.^1^, ^2^ Subsequently, the disease was discovered in many countries and swept the globe. The novel coronavirus, named the severe acute respiratory syndrome coronavirus 2 (SARS‐CoV‐2) by the International Committee on Taxonomy of Viruses, causes the coronavirus disease 2019 (COVID‐19), termed by the World Health Organization (WHO).^3^ COVID‐19, has a lower mortality rate, is more contagious and has caused a higher death toll than severe acute respiratory syndrome (SARS). Despite various measures being implemented to control the spread of COVID‐19, the number of confirmed cases is still increasing. By the end of October 8, 2021, according to the WHO, the outbreak of COVID‐19 has caused more than 236 million confirmed cases globally and more than 4.83 million deaths (https://covid19.who.int). Especially, early detection, diagnosis, and treatment of COVID‐19 patients are key points in the prevention and control of the epidemic situation.

In most cases, COVID‐19 patients were classified as asymptomatic, mild, moderate, and severe critical disease based on the clinical severity.^4^ Among these, the highly contagious of asymptomatic patients with a long latent period and strong infectivity made control and prevention efforts of COVID‐19 transmission extremely difficult.^5^ Laboratory testing plays a crucial role in early diagnosis, severe evaluation, and prognostic treatment of the disease. Currently, the nucleic acid testing depending on the real‐time PCR (RT‐PCR) remains the gold standard for the diagnosis of SARS‐CoV‐2‐infected patients. However, due to some limitations in nucleic acid testing, such as long detection cycle, expensive reagents and high requirements for sampling, and technicians and laboratory conditions, many COVID‐19 cases are not diagnosed accurately and timely.^6^, ^7^ Up to now, 369 cases of asymptomatic infection in China were still under medical observation, including 352 cases imported from abroad.^8^ Therefore, there is an urgent need to explore a rapid, simple, and feasible method for the diagnosis and screening of COVID‐19 patients, especially the asymptomatic cases.

As generally known, serum immunoglobulin M (IgM) antibodies appear in the early stages of viral infection, followed by the production of serum immunoglobulin G (IgG) antibodies, which are essential for long‐term immunity and immune memory. Furthermore, several studies have been reported that IgM and IgG antibodies can be detected in the first 1.5–8 days after the onset of symptoms, and may exist dynamically for a significant period of time.^9^, ^10^ Detecting serum IgM and IgG antibodies may provide a valuable detection method for the diagnosis and treatment of COVID‐19, especially, when screening for asymptomatic infection. The aim of this study was to provide evidence for screening infection in asymptomatic patients with COVID‐19 through the dynamic monitoring of IgM and IgG concentration levels.

## 2 MATERIALS AND METHODS

### 2.1 Patients

To identify asymptomatic individuals, the Disease Control and Prevention (CDC) of Loudi in China screened a total of 1844 close contacts (mean age 38.2 ± 19.9 years old), including 1003 men (mean age 38.7 ± 19.9 years old) and 841 women (mean age 37.7 ± 20.0 years), with 76 confirmed patients with COVID‐19 from January to May 2020. Nasopharyngeal swabs were collected for SARS‐CoV‐2 nucleic acid detection from all close contacts. Participants then submitted to a 14‐day medical quarantine observation period. Asymptomatic patients whose two consecutive nucleic acid tests were negative after the expiration of medical quarantine were observed at home for an additional 14 days. Asymptomatic cases were defined by a positive result from SARS‐CoV‐2 nucleic acid detection of respiratory tract specimens or serum‐specific IgM antibodies without corresponding clinical symptoms of COVID‐19 in close contacts, based on the diagnostic criteria for COVID‐19 and the requirements of the treatment plan (trial version 6 and trial version 7) issued by the National Health Commission of the People's Republic of China and National Administration of Traditional Chinese Medicine.^11^, ^12^

Twenty‐six cases without clinical symptoms within 28 days of observation were recruited from close contacts following a positive SARS‐CoV‐2 nucleic acid detection. Close contacts whose nucleic acid test results were negative continued to be isolated for an additional 14 days of observation. Fifty healthy individuals from the First Hospital of Hunan University of Chinese Medicine served as the control group. Age and gender distribution did not differ between the study group and the control group (*p* > 0.05). The detailed information of cases is presented in the results section. This study was approved by Ethics Committee of the First Hospital of Hunan University of Chinese Medicine.

### 2.2 Methods

The SARS‐CoV‐2 nucleic acid extraction reagent (Magnetic bead method, Jiangsu Master Biotechnology Co., LTD., item No: SDK60104) was used to extract viral nucleic acid with the aid of the automatic nucleic acid extraction instrument (Jiangsu Master Biotechnology Co., Ltd, item No: SSNP‐3000A). Open reading frame 1ab (ORF1ab) and nucleocapsid (N) gene of the dual nucleic acid detection kit was used for SARS‐CoV‐2 nucleic acid reverse transcription and amplification reagents (Shanghai Huirui Biotechnology Co., LTD, product no.: vr‐ii‐120). Interpretation criteria: a cycle threshold (Ct) value ≥39.2 means the genes to be tested were negative; a Ct value <35 means the gene to be tested was positive; Ct value between 35 and 39.2 indicate that the results are unclear and the test should be repeated. Once repeated, if the Ct value ≥39.2, the result was negative; however, if the Ct value <39.2, the result was positive. The SARS‐CoV‐2 antibody detection kit (colloidal gold method, Guangzhou Wanfu Biotechnology Co., LTD) and SARS‐CoV‐2 IgM and IgG detection kit (chemiluminescence method; Shenzhen Ya Huilong Biotechnology Co., LTD) were used for serum antibody assays, and a IFlash3000 automatic chemiluminescent immune analyzer was used (Shenzhen Ya Huilong Biological Technology Co., LTD). Interpretation criteria: samples with IgM and IgG concentrations <10.0 AU/ml are non‐reactive (negative), while concentrations ≥10.0 AU/ml are reactive (positive). All tests were conducted based on the manufacturer's instructions for all these kits.

### 2.3 Detection of SARS‐CoV‐2 nucleic acid in close contacts

Nasopharyngeal swabs were collected from 1844 close contacts and analyzed using a SARS‐CoV‐2 nucleic acid detection kit, following the manufacturer's instructions.

### 2.4 Qualitative detection of serum SARS‐CoV‐2 antibodies in asymptomatic patients

Blood was drawn from asymptomatic patients and centrifuged for 10 min to obtain serum. We qualitatively detected SARS‐CoV‐2 antibody using the colloidal gold method.

### 2.5 Quantitative and dynamic detection of serum SARS‐CoV‐2 IgM‐ and IgG‐specific antibodies in asymptomatic patients

Pharyngeal swabs were collected for SARS‐CoV‐2 nucleic acid tests until tests were negative for three consecutive times. Blood was collected from the first nucleic acid negative date (0 week) within weekly periods: ≤1 week, 1–2 weeks, 2–3 weeks, 3–4 weeks, 4–5 weeks, 5–6 weeks, and 6–7 weeks. Blood was centrifuged at 1650 *g* for 10 min. Sera were collected to detect IgM and IgG levels quantitatively through chemiluminescence.

### 2.6 Statistical analysis

Statistical analyses were performed using SPSS (v. 19.0) software with α of 5%. The measurement data are presented as mean ± standard deviation or median (interquartile range [IQR] P25, P75). Comparisons between two groups were performed using a t test and Wilcoxon's rank sum test. Enumeration data are expressed as percentages using the χ^2^ test.

## 3 RESULTS

### 3.1 Epidemiology of close contacts

**FIGURE 1:**
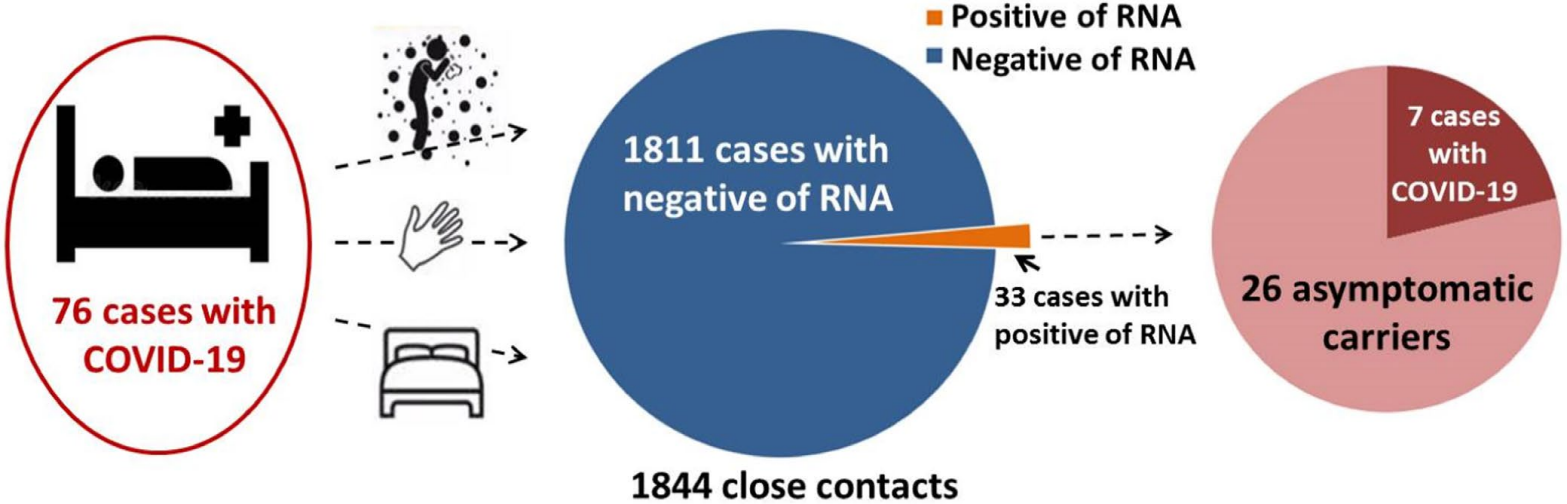
Epidemiological survey of close contacts with COVID‐19

Among the 1844 close contact cases, the nucleic acid swabs for 33 individuals with no clinical symptoms were positive; these individuals were treated as asymptomatic infected patients and were placed under medical observation. One week later, 7 of them developed fever, cough, and other symptoms (Figure 1, Table 1), confirming that they had contracted COVID‐19.

**TABLE 1:** Details of the 7 asymptomatic cases converted to confirmed patients

Cases	Sex	Age (year)	First day of positive nucleic acid	Date of symptoms onset	Symptoms and signs	Results of laboratory at the day of symptoms onset	First day of negative nucleic acid	Discharge date
1	M	14	2/6/2020	2/7/2020	Slight cough, no fever or other discomfort	Troponin slightly elevated, the rest normal	2/10/2020	2/12/2020
2	F	47	2/1/2020	2/3/2020	Cough, CT pulmonary infection on February 17th	ESR 87 mm/h↑, CRP 135.6 mg/L↑, WBC 11.31 × 10^9^/L↑; NEUT% 75.6%↑; LYMPH% 18%↓	2/9/2020	2/18/2020
3	M	13	2/1/2020	2/7/2020	Slight runny nose; chest CT: ground glass shadows in the right middle lung and left lower lung with a small amount of pericardial effusion.	ESR 13 mm/h↑	3/7/2020	3/8/2020
4	M	24	2/7/2020	2/9/2020	Occasional cough	PCT 0.62 ng/ml↑; cTnT 0.93 ng/ml↑	2/13/2020	2/14/2020
5	F	13	2/7/2020	2/10/2020	Rash on the hand	PCT 0.56 ng/ml; cTnI 0.39 ng/ml↑; ESR 12 mm/h↑	2/11/2020	2/13/2020
6	F	50	2/7/2020	2/10/2020	Fatigue, without chills and fever; Chest CT: small patchy shadows in the hilum	WBC 6.19 × 10^9^/L; NEUT% 73.31%; LYMPH% 21%; ESR 34 mm/h↑	2/11/2020	2/14/2020
7	F	47	2/7/2020	2/9/2020	Cough and anhelation	CRP 25.66 mg/L↑; ESR 61 mm/h↑; WBC 6.19 × 10^9^/L; NEUT% 78.4%↑, LYMPH% 14.7%↓; ESR 84 mm/h↑	2/11/2020	2/15/2020

Abbreviations: CRP, C‐reactive protein; cTnI, troponin I; cTnT, troponin T; ESR, erythrocyte sedimentation rate; LYMPH%, lymphocyte ratio; NEUT%, neutrophil ratio; PCT, procalcitonin; WBC, white blood cell.

The remaining 26 positive cases were defined as asymptomatic cases. Their average age was 33.3 0± 18.9 years, ranging from 3 to 77 years old. The average age of the 15 men was 34.2 ± 17.6 years old, and that of the 11 women was 32.1 ± 21.4 years old, there was no significant difference between sexes (*p* > 0.05). Furthermore, there was no significance in age between the 26 asymptomatic cases and the 7 patients with COVID‐19, which was 29.7 ± 17.5 years old (*p* > 0.05).

The time interval from the positive nucleic acid test to the negative result for the first time was defined as the communicable period, which ranged from 1–31 days, and the median communicable period (MCP) was 7.5 days.

### 3.2 Qualitative detection of antibodies to identify asymptomatic infections

Blood was drawn from 26 asymptomatic infected cases for nucleic acid tests with positive results. Twenty‐three of the 26 asymptomatic cases were positive for COVID‐19‐specific antibodies by the colloidal gold method, and only 3 cases were negative. The total positive rate of the colloidal gold method was 88.5% (23/26), which was significantly higher than that of the healthy control group (2.0%) (χ^2^ = 59.182, *p* < 0.001). The positive antibody rate between the men (86.7%) and women (90.9%) for asymptomatic infection was not significantly different (*p* > 0.05).

### 3.3 Quantitative dynamic changes in serum SARS‐CoV‐2 antibody in asymptomatic cases

**FIGURE 2:**
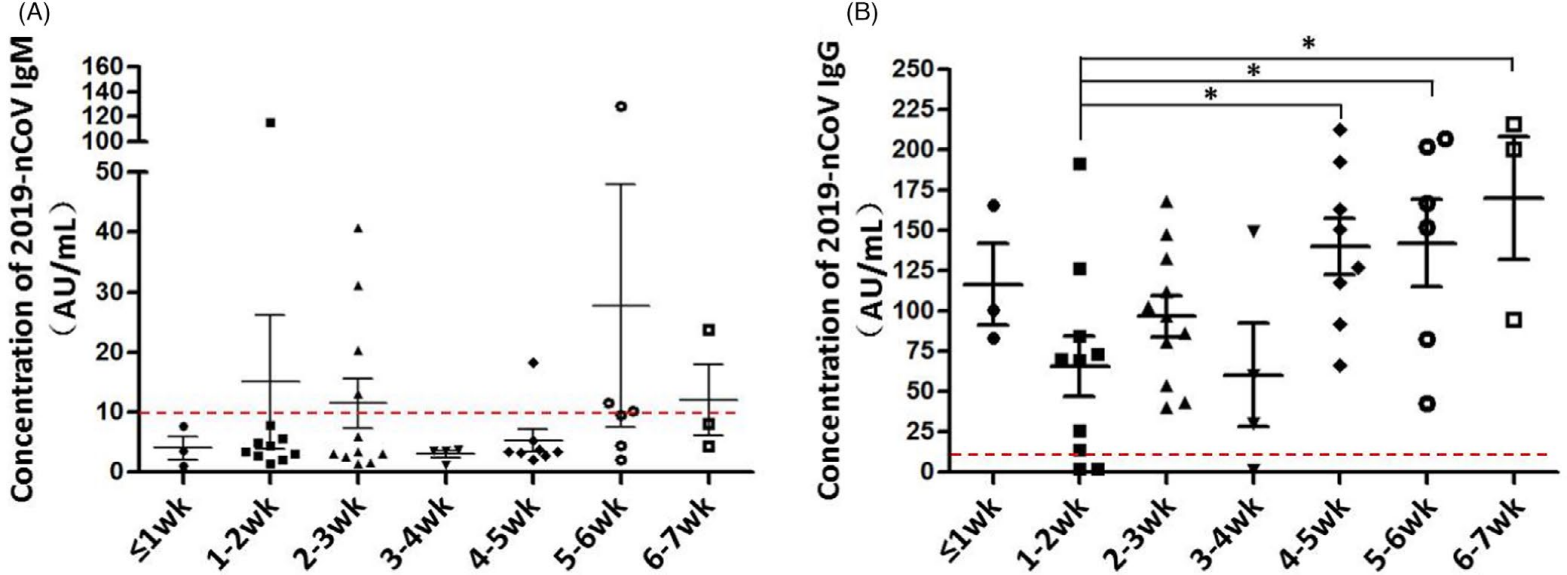
Changes in serum 2019‐nCoV IgM (A) and IgG (B) concentrations with time after 2019‐nCoV nucleic acid turned negative in 15 patients with asymptomatic novel coronavirus infection (Note: Abscissa W represents wk, red dotted line is the dividing line between negative and positive)

To further detect serum SARS‐CoV‐2‐specific IgM and IgG antibodies, 17 cases were screened from 26 asymptomatic patients to detect viral nucleic acid several times by monitoring on dynamic changes in antibody levels. The ages of the 17 asymptomatic cases, (men: n = 11 and women: n = 6) ranged from 8 to 77 years old, with an average of 31.5 years (IQR 14.5–45.5). The comparison of SARS‐CoV‐2 antibody levels between groups at different time periods (0–7 week) after nucleic acid negative transformation gave the following results:

There was no significant difference in IgM concentration between time points tested (0–7 weeks) after nucleic acid conversion (*p* > 0.05) (Figure 2A). However, a significant difference was observed in IgG concentrations with time changed (χ^2^ = 14.034, *p* = 0.029) (Figure 2B). The levels of serum IgM and IgG antibodies at different time periods (0–7 weeks) after nucleic acid negative conversion are shown in Table 2.

**TABLE 2:** Concentration of SARS‐CoV‐2 IgM and IgG between time points tested (0–7 week) after nucleic acid conversion

Weeks	N (total 46)	SARS‐CoV‐2 IgM	SARS‐CoV‐2 IgG
≤1 week	3	3.5 (2.3, 5.6)	100.5 (91.7, 133.3)
1–2 weeks	10	3.4 (2.7, 4.5)	73.3 (71.4, 78.8)
2–3 weeks	11	5.9 (4.7, 23.3)	102.6 (94.5, 107.1)
3–4 weeks	4	3.5 (2.3, 3.6)	60.0 (44.9, 104.9)
4–5 weeks	8	3.5 (2.8, 4.4)	126.9 (96.7, 169.6)
5–6 weeks	7	4.4 (3.3, 8.0)	152.0 (117.2, 177.0)
6–7 weeks	3	7.9 (6.1, 15.8)	200.3 (147.5, 207.9)

Serum IgG levels were significantly higher at 4–5 weeks (Z = −2.399, *p* = 0.016), 5–6 weeks (Z = −2.049, *p* = 0.040), and 6–7 weeks (Z = −2.197, *p* = 0.028) compared with those at 1–2 weeks after negative nucleic acid conversion. The serum IgG levels at 4–5 weeks of negative nucleic acid conversion were significantly higher than those at 3–4 weeks (*Z* = −2.038, *p* = 0.042).

Among the 17 asymptomatic cases, 46 times of dynamic detection in total were performed on SARS‐CoV‐2 antibodies after the nucleic acid turned negative, including 10 times of positive results in IgM and 43 times of positive results in IgG (Figure 2). The positive detection frequencies of IgM and IgG were 21.7% (10/46) and 93.5% (43/46), respectively. Among the 50 healthy individuals, only one case was IgM positive with a concentration of 17.5 AU/ml, and all IgG antibodies were negative. The positive detection rates of IgM and IgG in healthy individuals were 2.0% (1/50) and 0% (0/50), respectively.

The positive rates of IgM and IgG in the asymptomatic group within 7 weeks of negative nucleic acid conversion were significantly higher than those in the control group (χ^2^ = 84.660, *p* < 0.001 and χ^2^ = 9.201, *p* = 0.002, respectively) (Table 3).

**TABLE 3:** Positive results of serum IgM and IgG in 17 asymptomatic cases and 50 healthy controls after nucleic acid negative conversion

	SARS‐CoV‐2 IgM			SARS‐CoV‐2 IgG		
	+	−	Positive rate	+	−	Positive rate
Asymptomatic patients	10	36	21.7%	43	3	93.5%
Healthy individuals	1	49	2.0%	0	50	0%
Statistics	χ^2^ = 9.201, *p* = 0.002			χ^2^ = 84.660, *p* < 0.001		

## 4 DISCUSSION

Cutting off the route of transmission is an important preventive measure for infectious diseases. However, it has been reported recently that asymptomatic carriers can lead to person‐to‐person transmission in a community due to neglecting, thus posing a considerable challenge for the prevention and control of COVID‐19. Therefore, the proportion of asymptomatic infections needs to be determined in a timely manner through proper laboratory techniques.

There are two categories of asymptomatic patients with COVID‐19: infected individuals with positive viral nucleic acid test results but no recognizable symptoms and signs after 14 days of observation, and asymptomatic infections within the incubation period. As shown here, the nucleic acid test results of the infected individuals could be positive, even without self‐perceived or clinically recognizable symptoms and signs during sampling; however, clinical manifestations could appear later.

It was reported that a 20‐year‐old female asymptomatic patient infected five other members of her family after returning to Anyang, Henan province, China, on January 10, 2020.^13^ The five family members developed symptoms (fever and cough) and were diagnosed with COVID‐19. Nevertheless, the asymptomatic patient remained free of clinical symptoms, her C‐reactive protein and chest computed tomography (CT) scan were normal. With the emergence of increased cases from abroad, the asymptomatic SARS‐CoV‐2 populations have attracted more attention as a hidden source of infection.

Identifying asymptomatic infected populations early and accurately is essential for epidemic prevention and control. Here, we provide evidence by screening the infection in 26 asymptomatic individuals through dynamic analysis of IgM and IgG antibodies. Our results will provide a laboratory basis for understanding the status of the immune system and the pattern of specific antibody production. In this study, 33 asymptomatic cases with positive nucleic acids were screened, and seven of them were converted to confirmed COVID‐19 cases following development of fever and cough and changes in chest CT imaging during the period of medical quarantine and observation; the remaining 26 remained asymptomatic. However, continuous quantitative monitoring of serum antibodies was not performed for these seven patients. Thus, it is impossible to compare and analyze the characteristics of antibodies between asymptomatic and confirmed patients. In contrast to other viral pneumonia, recovering of COVID‐19 patients may need better care because they may have poor immunity and nutritional status.^14^ Remarkably, numerous studies have demonstrated that dynamically monitoring of IgM and IgG in serum was critical for COVID‐19 confirmed patients, which can assist nucleic acid detection to provide valuable guidance for the diagnosis, staging, and prognosis of SARS‐CoV‐2 infection.^9^, ^15^

To further evaluate the production of SARS‐CoV‐2‐specific antibodies in asymptomatic patients, blood specimens were collected regularly to detect the levels of serum SARS‐CoV‐2‐specific IgM and IgG antibodies after negative nucleic acid conversion. The positive rate of IgM antibodies in asymptomatic individuals was significantly higher even after negative nucleic acid conversion than that in healthy people. However, there was no significant difference in changes within 7 weeks after negative nucleic acid conversion (*p* < 0.05). Our previous study showed that the positive rate of IgM in patients with COVID‐19 was 75.9%.^16^ These results suggest that IgM may be a useful target for screening cases previously infected by SARS‐CoV‐2 and healthy people. Furthermore, IgM in some asymptomatic individuals after negative nucleic acid conversion is not easily degraded within 7 weeks.

According to our results, the serum concentration of IgG in asymptomatic individuals after the negative nucleic acid conversion was above normal reference and increased with time in the 7 weeks of observation. The positive rate of serum IgG in the asymptomatic group was 93.5% during 7 weeks after negative nucleic acid conversion. Long et al.^17^ reported that the positive rate of serum IgG in patients with acute phase COVID‐19 was 18.9%, and that of patients with the convalescent‐phase of COVID‐19 was 60.0%. Through these data, we infer that the IgG level of asymptomatic cases increased after negative nucleic acid conversion compared with the disease and recovery period. In addition, it may also be related to our longer observation time after the nucleic acid turned negative in asymptomatic cases, when the protective antibody IgG level began to rise.

To date, studies have showed that SARS‐CoV‐2 IgM and IgG antibodies detection methods had high specificity and sensitivity, and combined detection was superior to individual antibody detection. Interestingly, a recent study by Hu et al.^18^ found that heat‐inactivated serum at 56°C for 30 min can affect the level of SARS‐CoV‐2 antibody in serum, which may lead to false‐negative results. Additionally, Xiang et al.^19^ showed that the sensitivity and specificity of IgM were 77.3% and 100%, respectively, and the sensitivity and specificity of IgG were 83.3% and 95.0%, respectively, during detection of antibodies in COVID‐19 patients. Our previous study showed that the sensitivity and sensitivity of IgG (90.5% and 99.3%, respectively) were higher than those of IgM (75.9% and 94.0%, respectively) in COVID‐19 patients.^16^ Taken together, this indicates that the positive rate of IgM in asymptomatic patients after nucleic acid negative conversion is significantly lower than that in confirmed patients, whereas IgG remains at the same level as that of confirmed patients. Unfortunately, we were unable to obtain serum specimens from asymptomatic patients when their nucleic acid was positive, and thus it was not possible to compare changes in antibody levels between nucleic acid positive and negative.

In conclusion, nucleic acid testing, though time consuming and susceptible to sampling errors, is recommended to be the main basis for the diagnosis of asymptomatic patients. Antibody detection holds great value in the diagnosis and identification of asymptomatic patients because it is fast and convenient, and sampling can be easily standardized.

## CONFLICT OF INTEREST

The authors declare no conflicts of interest.

## Data Availability

All data are presented in this manuscript without additional supplementary information.
